# O6.1. HIPPOCAMPAL VOLUME IN ADOLESCENTS WITH PERSISTENT PSYCHOTIC EXPERIENCES: A LONGITUDINAL POPULATION-BASED MRI STUDY

**DOI:** 10.1093/schbul/sby015.222

**Published:** 2018-04-01

**Authors:** Ana Calvo, Erik O’Hanlon, Helen Coughlan, Ian Kelleher, Mary Clarke, Mary Cannon

**Affiliations:** 1 Universidad Internacional de La Rioja; 2 Royal College of Surgeons in Irel

## Abstract

**Background:**

Individuals with schizophrenia show significant brain morphological abnormalities. The ENIGMA consortium identified that patients with schizophrenia had smaller hippocampus, amygdala, thalamus, accumbens and intracranial volumes.1 Reduced hippocampal volume is one of the most consistent findings in schizophrenia research.2–4 Also, Previous research has reported differences in hippocampal volume and white matter integrity in young adolescents who report psychotic experiences.5,6 However there has been little longitudinal research to investigate the developmental trajectory of these regions in adolescence with an increased susceptibility to psychotic disorders.

**Aims:**

to investigate two-year longitudinal changes in hippocampal volume in a sample of adolescents who reported psychotic experiences relative to their peers. To investigate the role of presence of co-morbid DSM IV mental disorders and stressful life events in influencing hippocampal volume and study the differences in hippocampus volume between adolescents who were having persistent symptoms versus adolescents with remitting symptoms.

**Methods:**

A longitudinal case-control study of 50 community-based adolescents aged 13–16 years (25 with psychotic experiences and a matched sample of 25 without psychotic experiences), compared hippocampal volume. All participants were assessed at baseline and two years follow up. T1 weighted anatomical high-resolution imaging and high angular resolution diffusion imaging data were used to conduct quantitative anatomical volumetric evaluations of global hippocampal volume. Clinical interviews also provided information on psychotic experiences, co-morbid disorders and adverse life events.

**Results:**

There were significant differences in the Right and Left Whole hippocampus between PE and Control group at baseline and 2-year follow up (p≤ 0.05). There were significant differences between PE persist and Control group in the left and right whole hippocampus (p≤ 0.05).

**Discussion:**

The differences identified in our study suggest that early hippocampal reductions, may play a role in increasing vulnerability to psychosis.

**References:**

1. van Erp TG, Hibar DP, Rasmussen JM, et al. Subcortical brain volume abnormalities in 2028 individuals with schizophrenia and 2540 healthy controls via the ENIGMA consortium. Mol Psychiatry. 2016;21(4):585.

2. Wright IC, Rabe-Hesketh S, Woodruff PW, David AS, Murray RM, Bullmore ET. Meta-analysis of regional brain volumes in schizophrenia. Am J Psychiatry. 2000;157(1):16–25.

3. Nelson MD, Saykin AJ, Flashman LA, Riordan HJ. Hippocampal volume reduction in schizophrenia as assessed by magnetic resonance imaging: a meta-analytic study. Arch Gen Psychiatry. 1998;55(5):433–440.

4. Stein JL, Medland SE, Vasquez AA, et al. Identification of common variants associated with human hippocampal and intracranial volumes. Nat Genet. 2012;44(5):552–561.

5. Drakesmith M, Caeyenberghs K, Dutt A, et al. Schizophrenia-like topological changes in the structural connectome of individuals with subclinical psychotic experiences. Hum Brain Mapp. 2015;36(7):2629–2643.

6. Satterthwaite TD, Wolf DH, Calkins ME, et al. Structural Brain Abnormalities in Youth With Psychosis Spectrum Symptoms. JAMA psychiatry. 2016;73(5):515–524.

